# Addressing the Burden of Asynchronous Work to Promote Clinician Retention in Ambulatory General Internal Medicine Practices

**DOI:** 10.1007/s11606-025-09919-3

**Published:** 2025-11-12

**Authors:** Robert J. Doolan, Brittney R. Fraumeni, Lisa M. Schilling, Lauren A. Drake, Gena Weir, Anna Furniss, Mark A. Earnest

**Affiliations:** 1 Department of Medicine, Division of General Internal Medicine, University of Colorado School of Medicine, Aurora, CO USA; 2 Department of Psychiatry, University of Colorado School of Medicine, Aurora, CO USA; 3 Adult and Child Center for Outcomes Research and Delivery Science (ACCORDS), Department of Medicine, University of Colorado School of Medicine, Aurora, CO USA

**Keywords:** asynchronous work, physician, burnout, physician, primary care, changing schedule templates, dedicated telehealth

## Abstract

**Background:**

The growing burden of asynchronous work—such as managing messages, test results, and care coordination—has contributed to clinician burnout and attrition in primary care.

**Objective:**

To develop and evaluate scheduling templates that allocate time for asynchronous work during clinic sessions and assess their impact on clinicians, patients, and clinical operations.

**Design:**

We conducted a two-phase Plan-Do-Study-Act (PDSA) quality improvement initiative at four academic internal medicine clinics in metropolitan Denver. PDSA1 tested five alternative templates with asynchronous work blocks over 3 months for feasibility. PDSA2 evaluated the preferred template over 6 months for effects on clinician and patient satisfaction, clinician career plans, clinical quality, productivity, and access.

**Participants:**

In total, 27 attending physicians and APPs volunteered for the intervention group in PDSA1; 43 in PDSA2.

**Intervention:**

Clinical schedule templates with blocked time for non-patient-facing work.

**Main Measures:**

Survey items on career plans and clinician satisfaction, A1C and hypertension control, breast cancer screening, patient satisfaction, wRVUs, visit volume, and access (appointments made within 14 days).

**Key Results:**

Clinicians in PDSA1 favored a template with asynchronous time embedded in regular sessions with staff support present. Evaluating the preferred schedule template in PDSA2, clinician satisfaction improved (+ 2.25 on a −3 to + 3 scale), and the intention to continue clinical practice effort rose (from 8/37 to 20/34). Clinical quality and patient satisfaction remained stable. Visit volume dropped (−18.3 visits/EffcFTE/month, *p* < 0.01), but wRVUs were unaffected (−3.2 wRVUs/EffcFTE/month, *p* = 0.89). Access declined for both intervention and reference groups (−10% vs. −4%). A focus group emphasized enhanced clinician autonomy and control as key benefits.

**Conclusions:**

Integrating asynchronous work time into clinic schedules appeared to improve clinician satisfaction and retention without compromising care quality or wRVU productivity, while visit volume and access saw modest declines. This approach may support primary care workforce stability amid increasing asynchronous demands.

## Introduction

The Division of General Internal Medicine (GIM) at the University of Colorado faced significant clinician attrition in ambulatory clinics following the COVID-19 pandemic. Between February 2021 and July 2022, GIM recruited and onboarded four primary care providers (PCPs), adding 2.6 clinical full-time equivalents (cFTE). However, this could not offset the loss of 4.49 cFTE during the same period.

The primary cause of this loss, based on faculty interviews, was an increase in asynchronous work—tasks not tied to scheduled patient visits, such as responding to patient messages, following up on lab results, and reviewing medication requests. Clinicians also had to coordinate with care team members, including nurse care managers, clinical pharmacists, mental health therapists, and social workers. These challenges have been observed nationally.

A 2019 study showed that a full-time primary care clinician managed 390 in-basket tasks per week or 17,542 in-basket tasks per year.^1^ Such measures likely underestimate the work involved, as a single in-basket task, for example, receiving a patient’s blood pressure readings, may require multiple actions such as comparing results with previous readings, evaluating the treatment plan, and scheduling a follow-up visit. The COVID-19 pandemic accelerated the problem. One study showed a nearly 20% increase in inbox messages over the course of a single year.^2^ Most clinicians address the growing amount of asynchronous work without any change to the amount of care they provide via synchronous visits and with no protected time for the additional work.^3^ This burden is not evenly distributed. PCPs receive more messages than other clinician types,^4^ and general internal medicine clinicians receive more messages than other primary care specialists.^5^

This added work is not without consequence. PCPs have among the highest rates of burnout across clinical specialties. Approximately half of the PCPs in the USA report high burnout levels and the use of EMRs has been cited as a primary driver.^6^ These trends have a direct effect on the primary care workforce and may threaten the survival of primary care. To manage the workload, many ambulatory clinicians opt to work part-time or join a concierge practice,^1^ further exacerbating the shortage of primary care clinicians, which according to one estimate, will number between 20,200 and 40,400 providers by 2036.^7^ For these reasons, Bodenheimer and Sinsky (2014) proposed the quadruple aim, emphasizing the importance of prioritizing clinician well-being.^8^

We struggled to maintain clinical FTE and hypothesized that dedicating in-clinic time for asynchronous work could better align workload with available time. Lacking clear evidence in the literature, we conducted a two-phase Plan-Do-Study-Act (PDSA) cycle to pragmatically test revised schedule templates. We evaluated clinician satisfaction and intent to maintain clinical effort, alongside impacts on quality measures, patient satisfaction, productivity, and access.

## Methods

### Setting

Four ambulatory teaching clinics within a single academic health system in metropolitan Denver. (Table 1)

**Table 1:** Clinic Characteristics

Clinic makeup as of October 2022 (PDSA1 start)					
Clinic	Faculty MD/DO	Faculty APP	Clinical FTE	Patients	Resident clinic
Internal Med 1	25	2	9.2	13,305	Yes
Internal Med 2	17	1	7.1	11,308	Yes
Internal Med 3	10	2	8.5	11,335	No
Women’s Health	7	2	5.5	8322	No

### Participants

The GIM Division Head and Associate Division Head for Clinical Affairs (GIM leadership) oversaw this project. GIM leadership convened a 14-member workgroup — including physicians, APPs, clinic and resident directors, a division administrator, and a data analyst — to assess the current state, define goals, design new schedule templates for PDSA1, and assess results from PDSA1 to inform the design of PDSA2. Clinical operations leaders, including clinic medical directors, practice administrators, and lead schedulers, provided feedback through PDSA1 and PDSA2.

Clinicians eligible for PDSA1 and PDSA2 included attending physicians (MD/DO) and advanced practice providers (NP/PA) with empaneled patients, regardless of cFTE or seniority, who joined the division before October 1, 2021 (PDSA1), or March 1, 2023 (PDSA2). Resident physicians were excluded per GME guidelines governing patient volumes.

Intervention group enrollment was voluntary, with assurance that productivity changes would not affect compensation. In PDSA1, 27 of 57 eligible faculty participated (clinic breakdown: 9, 8, 7, 3); in PDSA2, 47 of 59 enrolled. Final PDSA2 analysis included 43 clinicians (19, 9, 12, 3), excluding two dropouts and two ineligible faculty (one practicing in an external clinic, one in GIM leadership). Non-participating and ineligible clinicians served as reference groups (PDSA1 34; PDSA2 18). Between PDSA1 and PDSA2, one eligible faculty member left, and three new faculty joined.

### Ethics

The study was deemed exempt by the Colorado Multiple Institutional Review Board. The project received approval at each stage from GIM, DOM, School of Medicine, and University of Colorado Hospital ambulatory leadership.

### Study Interventions

PDSA1 aimed to assess the feasibility and acceptability of five clinician schedule templates, all incorporating dedicated asynchronous work time with varying amounts, timing, and usage guidelines. Template 1 reserved 20–30 min of non-schedulable asynchronous work time per clinic session (3–4 h). Template 2 had five clinicians share space typically used by four (eight exam rooms), incorporating staggered 30-min blocks for asynchronous work and virtual visits to optimize room use. Template 3 allowed 20–30 min of asynchronous time per session, schedulable at the clinician’s discretion. Template 4 included 30 min of asynchronous work per in-clinic session plus one 4-h telehealth session per week. Template 5 provided a stand-alone 4-h asynchronous work session and a separate 4-h telehealth session per week.

**Figure 1:**
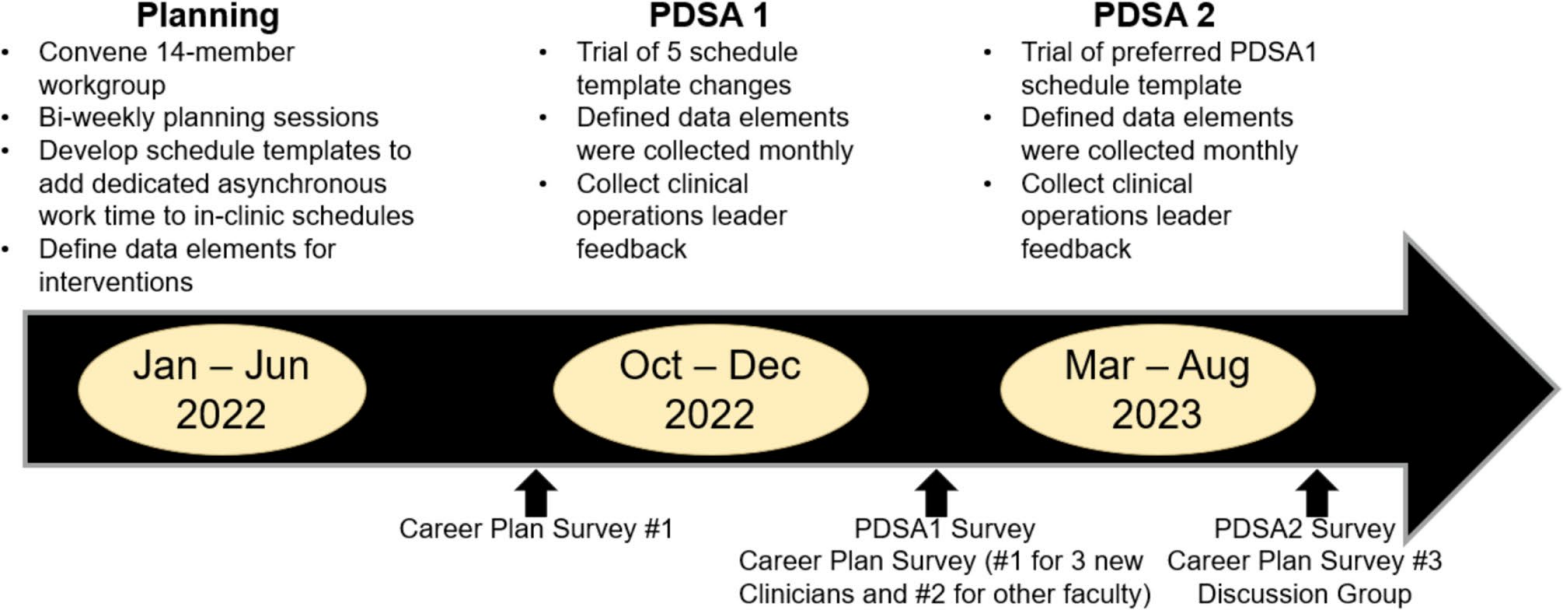
Project timeline.

After reviewing the outcomes of PDSA1, the workgroup selected Template 3 as the most promising schedule template to test in PDSA2 (Fig. 1).

### Evaluation

#### Data Collection and Measures: (see timeline Fig. 1)

Career plan survey (#1–3): Administered to intervention and reference group clinicians prior to PDSA1 and prior to PDSA2 for three new faculty, following PDSA1 and PDSA2. We asked respondents, “In the next one to three years, do you plan to make any adjustments in your clinical role?”

Clinician satisfaction surveys (PDSA1 and PDSA2 surveys): Administered to the intervention group after PDSA1 and again after PDSA2. We adapted six items from a validated clinician satisfaction survey^9^ with a 7-point Likert scale to assess the perceived clinical impact of the intervention compared to their perception of care provided prior to the intervention (ability to serve patients, timely response, connection with patients, providing best care, continuity of care, and managing asynchronous work).

Clinical operations leader feedback: Each week during both PDSA1 and PDSA2, clinical operations leaders were asked six questions: (1) What worked well? (2) What did not work well? (3) Have you noticed any positive impact on staff? (4) Have you noticed any negative impact on staff? (5) Did anything significant occur in your clinic in the past week outside of the pilot? (6) Are there other observations you would like to share?

Post PDSA2 discussion group: Following PDSA2, we held a recorded, open-format discussion with the intervention group to capture impacts not reflected in surveys.

Administrative data collection: For PDSA1, we compared monthly administrative data from October–December 2021 to the intervention period (October–December 2022); for PDSA2, from March–August 2022 to March–August 2023. Clinical quality was assessed via HbA1c and hypertension control and breast cancer screening rates, using data from the electronic medical record (EPIC). Patient satisfaction was measured using vendor-provided survey items (NRC Health): “Did this provider listen carefully to you?” “How likely would you be to recommend this facility to your family and friends?” Productivity was measured by clinician-generated work relative value units (wRVUs) and visit volume. Access was measured using a system-provided 14-day access measure: The number of new and return patient visits scheduled to occur within 14 calendar days of the appointment-made date, divided by all patient visits scheduled during the pre- and post-intervention periods.

### Analysis

#### Qualitative Analysis

 Qualitative data included comments from career plan surveys, PDSA1/PDSA2 surveys, and the post-PDSA2 discussion group, where transcripts were analyzed thematically using rapid qualitative analysis. Feedback from clinical operations leaders was reviewed in real time by the workgroup to identify operational challenges. All comments were reviewed and thematically coded by multiple reviewers, with discrepancies resolved by consensus.

#### Quantitative Analysis

We performed descriptive analyses on survey questions for clinicians completing career plan surveys 1–3, as well as the PDSA1 and PDSA2 surveys. We compared intervention and reference groups’ career plans survey #1 results to those after PDSA1 (#2) and PDSA2 (#3). Intervention group responses for each of the six clinician satisfaction measures from PDSA1 and PDSA2 surveys were aggregated. Pre-/post-statistical comparisons were not possible, as data were collected only post-intervention and only for the intervention group.

We assessed administrative data using a difference-in-difference analysis where a change of 2.5% was defined by the workgroup as meaningful for these measures.

We standardized wRVUs and visit volume by dividing by effective clinical FTE (EffcFTE), which represents the actual time a clinician spent in the clinic, accounting for time off and away from the clinic. We did not perform statistical analysis on PDSA1 wRVU and visit volume results due to low numbers of intervention clinicians, multiple template changes, and limited intervention duration. For PDSA2, we used generalized linear mixed models, adjusted for clinic-level factors, for statistical analysis. A *p*-value < 0.05 was considered significant. Data were analyzed using SAS version 9.4.

A sub-analysis was performed to test if the percentage of blocked asynchronous work time predicted a difference in wRVUs generated. The intervention design included known differences in asynchronous work time (20 or 30 min) and variability in session lengths (3–4 h across clinicians). We included PDSA 2 intervention group clinicians that did not introduce any dedicated telehealth time into their schedule template during the intervention (*n* = 33), as adding dedicated telehealth introduced a variable that could not be controlled for. Simple linear regression was used for analysis.

## Results

### Pre-Intervention

**Figure 2:**
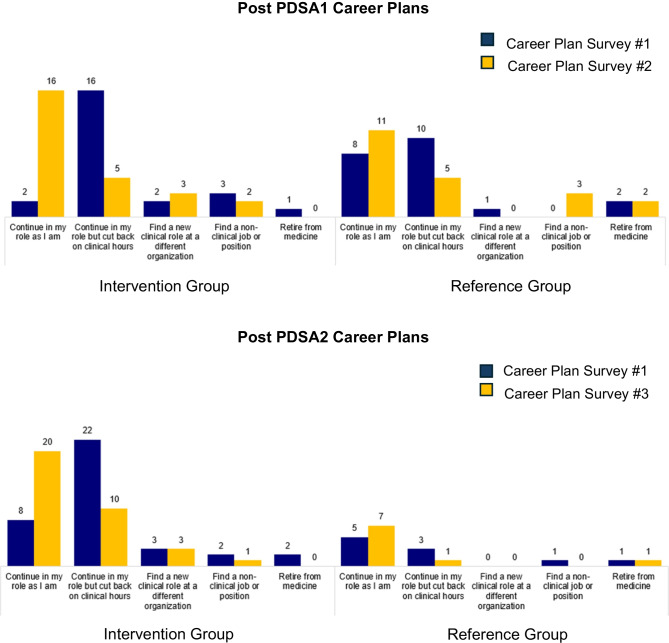
Future career plans: change in frequency of responses for the intervention and reference groups from career plan surveys #1 to #2 (Post PDSA1) or #3 (Post PDSA2).

In total, 45/57 (79%) of eligible faculty members completed career plan survey #1; 3/3 (100%) faculty members that joined the division prior to PDSA2 completed career plan survey #1 (Fig. 2).

### PDSA1

**Figure 3:**
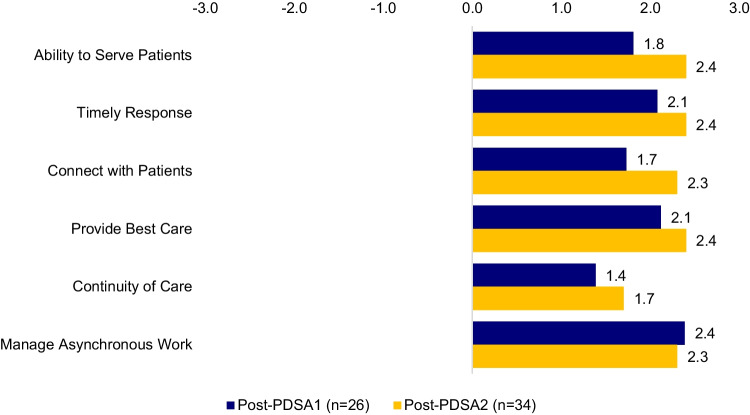
Clinician satisfaction: PDSA1 and PDSA2 survey results (intervention groups).

In total, 26 of 27 intervention group clinicians (96%) completed career plan #2 and PDSA1 surveys, and 21 of 34 reference group clinicians (62%) completed the career plan survey #2. The intervention group showed a positive shift in future career plans compared to the reference group (Fig. 2). Overall intervention clinician satisfaction results were positive (+ 1.92 on a −3 to + 3 scale) for all six measures of the PDSA1 survey (Fig. 3). Template 4 received lower ratings (mean 0.88), including negative scores for continuity of care (−0.8) and ability to serve patients (0).

Intervention group clinicians preferred asynchronous time integrated into clinic sessions with staff support present (as in templates 1–3), over isolated blocks (templates 4 and 5). Feedback from the intervention group and clinical leaders found template 2 operationally unfeasible due to workflow disruptions.

Clinical quality measure changes were comparable between intervention and reference groups (A1C −0.4% vs. +1.7%, hypertension +3.2% vs. +2.6%, breast cancer screening +4.6% vs. +5.9%). Patient satisfaction was similar between intervention and reference groups for, “Provider listened carefully” (+ 1.3% vs. +2%) but slightly exceeded the threshold for meaningful change in, “How likely would you be to recommend this facility to your family and friends favoring the intervention group” (+3.5% vs. +0.1%).

wRVUs adjusted for EffcFTE decreased on average from 1686 to 1498 for the intervention group, with no change noted for the reference group. However, the template 3 intervention group clinicians (5) demonstrated a year-over-year increase in wRVUs from 1486 to 1558. Visit volume declined 11–14% in templates 1, 4, and 5, with minimal impact (0 to −1%) in templates 2 and 3. A 1% increase was noted for the reference group. Access (appointments within 14 days) decreased across all template changes, as well as with the reference group (−12% vs. −6%).

We chose template 3 to advance to PDSA Cycle 2 as this model showed a positive change in clinician satisfaction and intention to maintain cFTE while maintaining clinical quality and patient satisfaction and minimally impacting wRVUs and visit volume.

### PDSA2

In total, 34 of 43 intervention group clinicians (79%) completed career plan #3 and PDSA2 surveys, and 9 of 18 reference group clinicians (50%) completed the career plan #3 survey. The intervention group showed a positive shift in future career plans compared to the reference group (Fig. 2).Intervention clinician satisfaction was positive for all six measures of the PDSA2 survey (+2.25 on a −3 to + 3 scale, Fig. 3).

Of the 43 intervention group clinicians invited to participate in post PDSA2 discussion groups, 16 (37%) attended. Clinicians uniformly reported a positive impact from the intervention (Table 2), highlighting the value of flexible, dedicated time to complete asynchronous work. They also described an increased sense of control that improved their overall well-being. While time remained insufficient to complete all clinical duties during regular work hours, the added flexibility was highly valued.

**Table 2:** Captured Themes and Representative Quotes from Focus Groups (*N*= 16)

Theme	Description of theme	Representative quote
Flexibility with asynchronous work time	Each day in clinic is unique, so having flexibility with the protected time, being able to use it as the day calls for, and not following rigid guidelines was perceived as being key to the success of and satisfaction with the intervention	“I have more time to get caught up with tasks…that may be answer phone calls, get notes done…in any case I have less to do at night after dinner.”
Ability to slow down and mentally decompress	Clinicians feel less rushed between tasks, spend additional time with patients without fear of being late for all remaining patients on their schedule, and have a break from the cognitive load of being a PCP	“Knowing that I will catch up and then get back on track… there's a sense of relaxation or peace coming into it that wasn't there before.”
Autonomy during clinical workday	Clinicians shared a collective view that they have a lack of control when it comes to scheduling, patient-related tasks, and organizational expectations. But having autonomy in how to use the protected time helped get a sense of control back into their workday	“Just giving us a tiny bit of control to manage our patients and our panel is huge, at least for me.”
Improved sense of humanity in clinic	Simple humane tasks that many people take for granted, such as having a bathroom break, a chance to refill a water bottle, chat with a colleague about work tasks, or even sit down for lunch are often not prioritized in a typical clinical schedule	“[Knowing] there's a break coming. I know I can go to the bathroom…I mean, that sounds really basic, but sometimes before…I would actually have to ask myself ‘where in my day am I taking a bathroom break?”
Time still needed for tasks	The addition of protected time does not allow for all asynchronous tasks to be completed during the clinical workday, particularly for clinicians that supervise residents or cover others’ patient panels	“There’s still more demand on our time than we have available.”

A1C and HTN control improved in the intervention and reference groups (A1C + 0.5% vs. + 3.7%, hypertension + 1.3% vs. + 4%), slightly exceeding the threshold for meaningful change favoring the reference group. Breast cancer screening rates were similar across both groups (+ 0.5% vs. −1%). Patient satisfaction remained similar between the intervention and reference groups (provider listened carefully + 4% vs. + 3%, how likely would you be to recommend this facility to your family and friends + 4% vs. 3%).

**Figure 4:**
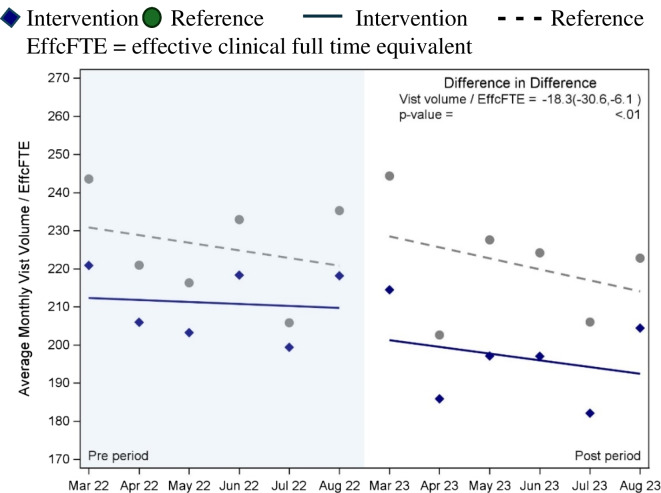
PDSA2 difference between intervention and reference groups for change in visit volume/EffcFTE* post- vs pre-intervention.

The change in wRVUs for the intervention adjusted for EffcFTE was not significantly different between the intervention and reference groups (−3.2 wRVUs/EffcFTE per month—95% CI −48.4, 42.0, *p* = 0.89). Figure 4 shows the change in visit volumes adjusted for EffcFTE pre- vs. post-intervention was significantly lower for the intervention group than the reference group (−18.31 visit volume/EffcFTE per month, *p* < 0.01). Access (appointments within 14 days) decreased for both intervention and reference groups (−10% vs. −4%).

**Figure 5:**
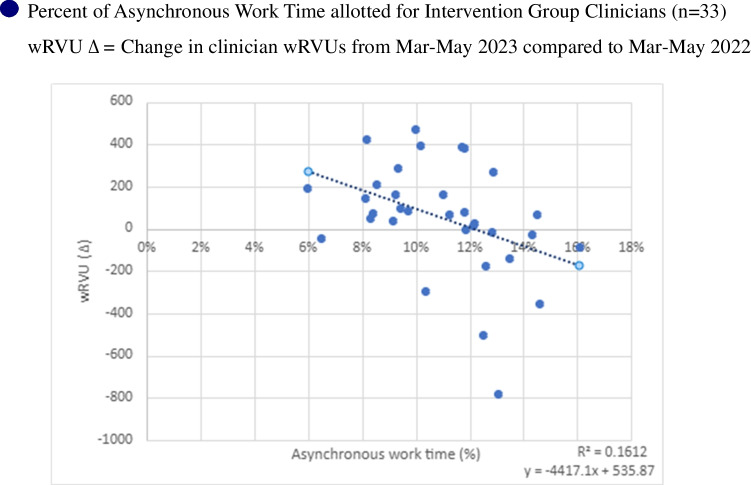
PDSA2 scatter plot of the change inwRVUs by % time blocked for asynchronous work.

Thirty-three intervention group clinicians were included in the wRVU sub-analysis. The percentage of asynchronous work time allotted predicted the wRVU change noticed in the intervention. The scatter plot and linear regression shown in Fig. 5 detail the impact. The fitted regression model was change in wRVUs = 535.87 +  −4417.12*(percent asynchronous work time). The regression was statistically significant (*R* squared = 0.16, *F *(1,31) = 5.96, *p* < 0.05). A loss in wRVUs begins to appear when the percentage of blocked asynchronous work time is greater than 12% of total clinical time.

## Discussion

Embedding asynchronous work time into clinic sessions shows promise for improving clinician satisfaction. Intervention clinicians reported a reduced likelihood of decreasing cFTE within 1–3 years. Impacts on quality, patient satisfaction, and wRVUs were minimal, with a modest negative effect on visit volume and access.

Post-intervention surveys and discussion group data suggest that much of the impact on clinician satisfaction came from an increased sense of control over their workday by providing clinicians flexibility in how the asynchronous work time was used and the value of providing this time when clinical team members were available. Loss of autonomy has been identified as a significant contributor to burnout,^10^ and based on this intervention, it appears that even a small change in clinician control can yield meaningful improvements in job satisfaction.

There are clear costs to this intervention in terms of productivity. On average, intervention group clinicians had approximately 12.5% of their clinical time blocked for asynchronous work, which would predict a 12.5% reduction in both visit volume and wRVUs. Visit volume, as noted in PDSA2, declined significantly; however, it was less than expected. As one possible explanation, clinician comments suggested that some of the dedicated asynchronous work time was used to see patients. The impact on wRVUs was also less than predicted, with the difference-in-difference analysis noting a slightly lower wRVU production for the intervention group that did not meet statistical significance. Our data suggests that a loss in wRVUs begins to appear when the percentage of blocked asynchronous work time is greater than 12% of total clinical time, while wRVUs increased above baseline when 6–10% of clinical time is blocked for asynchronous work. This suggests that there may be an optimal amount of asynchronous work time to maximize the intervention benefit while minimizing the impact on production. Other factors, such as a modest increase in the level of billing per encounter, could also help explain this finding. Defining this relationship more precisely will require additional research.

Patient access did decline with the intervention, which was expected. Patient satisfaction increased in all domains — a finding that could be an effect of care provided by more satisfied clinicians or because clinicians felt they could take more time with each patient. It could also be because the asynchronous time enabled clinicians to more often solve patient problems without requiring additional visits.

This study has limitations. As a quality improvement intervention within a single academic medical center, the study was designed to solve a major local problem rather than to achieve generalizability to other healthcare settings. Furthermore, while participation in the study surveys and focus group was high, overall representing a majority of clinicians in our division, the total number of respondents was small, limiting the usefulness of statistical analysis for those data sources. Similarly, while our administrative data analyses of patient-level data did support statistical investigation, we could not usefully account for potential confounders such as patient complexity. More research is needed in other settings with more clinicians to see if this strategy yields similar results.

Despite these limitations, however, this study provides important information for researchers and healthcare systems as a basis for future work. A strength of this study is that the problem we addressed is common and urgent, yet has eluded practical solutions within healthcare systems. We assessed both the acceptability of our interventions and potential negative impacts on productivity, supported by qualitative input from clinicians and clinical operations leaders.

The estimated downstream cost of losing a cFTE is $500–800,000 per year.^11^ Viewed in this manner, the value of avoiding cFTE reductions may outweigh the modest costs of investment in interventions like ours. A small loss of patient access or clinician productivity in the short term may be outweighed by the long-term access loss that can come from early retirement and cFTE reductions.

In summary, providing dedicated time during normal clinic hours to accomplish asynchronous work tasks appears to be a strategy to address some of the threats facing the primary care workforce. More study is needed to determine if the effects observed are preserved over time, if the stated intentions to continue clinical work are borne out, and if these findings are replicable in other settings.

## Declarations

### Clinical Relevance Statement

This study demonstrated a successful implementation of asynchronous work time in daily schedules for PCPs.  Readers should be able to implement similar methods within their health systems. 
